# Magnetization transfer and frequency distribution effects in the SSFP ellipse

**DOI:** 10.1002/mrm.28149

**Published:** 2019-12-24

**Authors:** Tobias C. Wood, Rui P. A. G. Teixeira, Shaihan J. Malik

**Affiliations:** ^1^ Department of Neuroimaging King's College London London UK; ^2^ School of Imaging Sciences and Biomedical Engineering King's College London London UK

**Keywords:** MT, PLANET, SSFP

## Abstract

**Purpose:**

To demonstrate that quantitative magnetization transfer (qMT) parameters can be extracted from steady‐state free‐precession (SSFP) data with no external *T*_1_ map or banding artifacts.

**Methods:**

SSFP images with multiple MT weightings were acquired and qMT parameters fitted with a two‐stage elliptical signal model.

**Results:**

Monte Carlo simulations and data from a 3T scanner indicated that most qMT parameters could be recovered with reasonable accuracy. Systematic deviations from theory were observed in white matter, consistent with previous literature on frequency distribution effects.

**Conclusions:**

qMT parameters can be extracted from SSFP data alone, in a manner robust to banding artifacts, despite several confounds.

## 1 INTRODUCTION

Quantitative magnetization transfer (qMT) imaging is of great interest for investigating multiple neuropathologies, particularly those relating to myelin.^1^, ^2^ The steady‐state free‐precession (SSFP) sequence is sensitive to MT and its short acquisition time is an advantage compared to more traditional gradient echo sequences.^2^, ^4^ qMT parameters can be found from SSFP data acquired at multiple flip angles,^5^ but this requires an additional *T*_1_ map and assumes that all voxels are on‐resonance. This is valid at 1.5T but not at 3T or above where susceptibility gradients produce banding artifacts.^6^

The PLANET method can calculate *T*_1_ & *T*_2_ from complex‐valued SSFP data at a single flip angle but multiple phase increments, provided TR is sufficiently long for MT effects to be insignificant.^7^, ^8^ We show that by combining these methods qMT parameters can be calculated from SSFP data at multiple flip angles and phase increments. However, we demonstrate that frequency distribution effects in white matter, where multiple pools of water protons exist, present a significant confound.^8^, ^9^, ^10^

## 2 THEORY

We incorporated MT effects into the SSFP signal ellipse by following^5^ but including off‐resonance effects. We make the derivation in terms of the bound pool fraction *f*_*b*_ = *M*_0*b*_/(*M*_0*f*_ + *M*_0*b*_) where the subscripts *f* & *b* denote the free and bound pools respectively. Hence the magnetization vector is $M\;=\;(M_{\mathrm{xf}},\;M_{\mathrm{yf}},\;M_{\mathrm{zf}},\;M_{\mathrm{zb}})$ as the bound pool is not visible during the MR experiment. We then define:

(1) $$\begin{array}{c} R\;=\;\left[\begin{array}{cccc} E_{2f} & 0 & 0 & 0\\ 0 & E_{2f} & 0 & 0\\ 0 & 0 & E_{1f} & 0\\ 0 & 0 & 0 & E_{1b} \end{array}\right]\;X\;=\;\left[\begin{array}{cccc} 1 & 0 & 0 & 0\\ 0 & 1 & 0 & 0\\ 0 & 0 & f_{f}+f_{b}E_{k} & f_{f}\left(1-E_{k}\right)\\ 0 & 0 & f_{b}\left(1-E_{k}\right) & f_{b}+f_{f}E_{k} \end{array}\right]\\ O\;=\;\left[\begin{array}{cccc} \cos\psi & \sin\psi & 0 & 0\\ -\sin\psi & \cos\psi & 0 & 0\\ 0 & 0 & 1 & 0\\ 0 & 0 & 0 & 1 \end{array}\right]\;P\;=\;\left[\begin{array}{cccc} \cos\alpha \cos\phi & -\sin\alpha \sin\phi & \sin\alpha & 0\\ \sin\phi & \cos\phi & 0 & 0\\ -\sin\alpha & \sin\alpha \sin\phi & \cos\alpha & 0\\ 0 & 0 & 0 & E_{w} \end{array}\right] \end{array}$$

 **R** represents relaxation, with *E*_1*b*_ =  exp (−*TR*/*T*1_*b*_), *E*_1*f*_ =  exp (−*TR*/*T*1_*f*_) and *E*_2*f*_ =  exp (−*TR*/*T*2_*f*_). **X** represents exchange, which is governed by *f*_*f*_, *f*_*b*_, the forward (bound‐to‐free) exchange rate *k*_*bf*_, the reverse (free‐to‐bound) exchange rate *k*_*fb*_ and the exponential average of the exchange rates *E*_*k*_ =  exp (−(*k*_*bf*_ + *k*_*fb*_)*TR*). **O** describes accrued off‐resonance phase *ψ* = 2*π*Δ*f*_0_ *TR*.

**P** represents the RF pulse, including excitation of the free pool by the angle *α*, rotation by a phase increment *ϕ*_*j*_, and saturation of the bound pool by the factor *E*_*W*_ =  exp (−*WT*_*RF*_). *T*_*RF*_ is the length of the RF pulse, and *W* is the saturation rate:

(2) $$W\;=\;\pi G_{0}\frac{p_{2}}{p_{1}^{2}}\frac{\alpha^{2}}{T_{\mathrm{RF}}^{2}}$$

 *G*_0_ is the value of the lineshape at resonance, *p*_1_ is the ratio of the pulse's mean *B*1 to the maximum *B*1, and *p*_2_ is the ratio of the mean *B*1^2^ to the maximum *B*1^2^.^11^, ^12^ The factors *p*_1_ and *p*_2_ describe the shape of the RF pulse relative to a block pulse, and provide a simple way to scale the saturation rate for different flip angles and pulse lengths (the saturation rate of a block pulse simply scales with flip angle squared and the inverse square of pulse‐length). We assumed a fixed value of *G*_0_ = 14 μs, which is the extrapolation of the Super‐Lorentzian from an off‐resonance value of 1 kHz to zero.^5^, ^13^ Strictly, the lineshape should be evaluated at the local off‐resonance value; however at 3T, the highest off‐resonance values in the human brain are approximately 100 Hz which is well below the 1 kHz value at which the Super‐Lorentzian is assumed to be flat. Finally, we define the following useful terms, similar to Equation 11 of Gloor et al^5^:

(3) $$\begin{array}{cc} A & \;=\;1-E_{w}E_{1b}\left(f_{b}+f_{f}E_{k}\right)\\ B & \;=\;f_{f}-E_{k}\left(E_{w}E_{1b}-f_{b}\right)\\ C & \;=\;f_{b}\left(1-E_{1b}\right)\left(1-E_{k}\right) \end{array}$$

We then define the magnetizations before and after the RF pulse as

(4) $$\begin{array}{cc} M_{n+1}^{-} & \;=\;X({O\mathrm{RPM}}_{n}^{-}+M_{0})\\ M_{n}^{+} & \;=\;{\mathrm{PM}}_{n}^{-}\\ M_{0} & \;=\;{\left[0,\;0,\;M_{0f}\left(1-E_{1f}\right),\;M_{0b}\left(1-E_{1b}\right)\right]}^{T} \end{array}$$

and then calculate the steady‐state solution at the echo time *TE* = *TR*/2:

(5) $$M_{\mathrm{ss}}\;=\;\sqrt{OR}P{\left(I-\mathrm{XO}\mathrm{RP}\right)}^{-1}{\mathrm{XM}}_{0}$$

where the term $\sqrt{OR}$ represents relaxation and accrued off‐resonance between the RF pulse and *TE*. The complex‐valued steady‐state magnetization is then:

(6) $$M_{\mathrm{xy}}\;=\;\frac{M_{0f}\left(B\left(1-E_{1f}\right)+C\right)\left(1-E_{2f}e^{-i\theta }\right)\sin\alpha \sqrt{E_{2f}}e^{i\psi /2}}{A-BE_{1f}\cos\alpha -E_{2f}^{2}\left(BE_{1f}-A\cos\alpha \right)-E_{2f}\left(A-BE_{1f}\right)\left(1+\cos\alpha \right)\cos\theta }$$

where *θ* = *ψ* + *ϕ*_*n*_. This can be converted to the elliptical form^6^

(7) $$M\;=\;\frac{M_{E}\left(1-ae^{i\theta }\right)}{1-b\cos\theta }\exp i(\psi /2+\phi_{\mathrm{RF}})$$

where

(8a) $$M_{E}\;=\;\frac{M_{0f}\sqrt{E_{2f}\left(B\left(1-E_{1f}\right)+C\right)\sin\alpha }}{A-BE_{1f}\cos\alpha -E_{2f}^{2}\left(BE_{1f}-A\cos\alpha \right)}$$

(8b) $$a\;=\;E_{2f}$$

(8c) $$b\;=\;\frac{E_{2f}\left(A-BE_{1f}\right)\left(\cos\alpha +1\right)}{A-BE_{1f}\cos\alpha -E_{2f}^{2}\left(BE_{1f}-A\cos\alpha \right)}$$

In the definition of *M*_*E*_, we have included the decay of transverse magnetization at *TE* via the $\sqrt{E_{2f}}$ term, but off‐resonance in the same time period is incorporated into Equation 7 so that *M*_*E*_ remains a real quantity. Equation 7 also contains the *B*_1_+ RF phase term *ϕ*_*RF*_, which was neglected earlier for simplicity.^7^

This equation has the same form as the elliptical expression of Xiang and Hoff, where *M*_*E*_ is the geometric solution, which is close to the geometric center of the ellipse.^6^ *a*, combined with *M*_*E*_, determines the ellipse size, and is purely affected by *T*_2_ and no MT parameters. *b* describes how flattened the ellipse is. In contrast to PLANET where *M*_0_, *T*_1_, and *T*_2_ can be algebraically calculated from *M*_*E*_, *a*, and *b*, it is not possible to extract *M*_0*f*_, *T*_1*f*_, *f*_*b*_, and *k*_*bf*_ from a single ellipse, principally because only two of the ellipse parameters (*M*_*E*_ and *b*) depend on MT. However, by acquiring multiple ellipses with different MT weightings, it is possible to recover the MT parameters.

## 3 METHODS

### 3.1 Model fitting

The following assumes multiple ellipses labeled *i* = 1 … *m* acquired with phase increments *j* = 1 … *n*. Each ellipse has a different *α*_*i*_ or *T*_*RFi*_ to change the MT weighting. Fitting proceeds in two steps. We first calculate the ellipse parameters for each ellipse separately, resulting in *m* values each of *M*_*Ei*_, *a*_*i*_ and *b*_*i*_. In the second step, the MT parameters are found from the ellipse parameters.

PLANET calculates *M*_*E*_, *a*, and *b* algebraically for a single ellipse, but is difficult to use in the region near the Ernst angle because of a sign change in the formula for *b*.^7^ At 3T and above, due to *B*_1_^+^ inhomogeneity, it cannot be guaranteed that *α* will be above the Ernst angle in all voxels. Also, although numerous algebraic ellipse methods exist, they have been developed for the general case where no a priori information is available,^14^, ^15^ but here the ellipse must be vertical (*b* < 2*a*/(1 + *a* ^2^)) and must not contain the origin.^6^ Hence in preference, we used a bounded nonlinear least‐squares fit of Equation 7 to find *M*_*E*_, *a*, and *b* for each ellipse, implemented in our freely available toolbox.^16^ The cost function was

(9) $$\rho_{\mathrm{Ellipse}}\;=\;\sum_{j\;=\;1}^{n}ℜ{\left(m_{j}-s_{j}\right)}^{2}+ℑ{\left(m_{j}-s_{j}\right)}^{2}$$

where *s*_*j*_ is the acquired complex‐valued SSFP data for *ϕ*_*j*_ and *m*_*j*_ is the corresponding value calculated from Equation 7. The bounds were 0 < *a* <  exp (−*TR*/5000), 0 < *b* < 1, −2*π* < *ψ* < 2*π*, and −2*π* < *ϕ*_*RF*_ < 2*π*, and we forced the ellipse to remain vertical. The initial guess for *M*_*E*_ was |∑*s*_*j*_/*n*|, while *a* and *b* were calculated assuming *T*_1*f*_/*T*_2*f*_ = 1000/50 ms for the specified *TR* and *α*. The initial guesses for the tightly coupled *ψ* and *ϕ*_*RF*_ were more complex. Three guesses for *ψ* (−*π*, 0, +*π*) were tried, and for each *ϕ*_*RF*_ was set to the phase of the complex mean of the data minus the guess for *ψ*. The guess with the lowest root‐mean‐square residual to the data was used as the initial guess for the full nonlinear least squares algorithm. A Huber Loss function was applied to the residuals to suppress the influence of outliers.^17^

From the set of *a*_*i*_ values, *T*_2*f*_ can then be found directly:

(10) $$T_{2f}\;=\;\frac{\sum_{i\;=\;1}^{m}-TR_{i}/\log a_{i}}{m}$$

To find the MT parameters, a nonlinear least squares fit was used with the cost‐function:

(11) $$\rho_{\mathrm{MT}}\;=\;\sum_{i\;=\;1}^{m}{\left(M_{\mathrm{Ei}}-M_{\mathrm{Ei}}^{\prime }\right)}^{2}+\sum_{i\;=\;1}^{m}{\left(b_{i}-b_{i}^{\prime }\right)}^{2}$$

where $M_{\mathrm{Ei}}^{\prime }$ and $b_{i}^{\prime }$ are found from Equations (8a) and (8c), respectively. Values of *M*_*Ei*_ were scaled by their mean to make them comparable in magnitude to *b*. An initial guess of *M*_0_ = 13, *f*_*b*_ = 5%, *k*_*bf*_ = 2 s^−1^ and *T*_1*f*_ = 1000 ms was empirically determined to converge for a majority of voxels in the brain. *T*_1*b*_ was set equal to *T*_1*f*_.^5^

### 3.2 Simulations

Monte Carlo simulations were conducted to validate our method. The tissue parameters were *M*_0*f*_ = 100, *T*_1*f*_/*T*_2*f*_ = 1100/80 ms, *k*_*bf*_ = 4.45 s^−1^, and *f*_*b*_ = 10%, and the sequence parameters were *α*_*i*_ = 15, 15, 30, 30^∘^, *T*_*RFi*_ = 256, 1024, 256, 1024 μs, and *TR*_*i*_ = 7, 7.768, 7, 7.768 ms, producing four MT weightings. The sequence parameters correspond to our in vivo protocol which was dictated by system limits that are discussed below.

*M*_*Ei*_, *a*_*i*_, and *b*_*i*_ were then calculated with Equation 8 and combined with random values of *ψ* and *ϕ*_*RF*_ drawn from uniform distributions between −*π* and *π*. *m*_*j*_ was calculated with Equation 7 and *ϕ*_*j*_ = *j*60^∘^, *j* = 1 … 6. This gives 24 complex datapoints (four ellipses with six points each) to which complex‐valued Gaussian noise with *σ* = *M*_0_/500 was added. This value corresponds to an SNR (defined as mean signal magnitude divided by noise) of approximately 70, which matches literature values for gradient echo sequences in white matter.^18^4,096 realizations of noise were used.

### 3.3 In vivo measurements

Images from a healthy volunteers were acquired on a GE MR750 3T scanner equipped with 50 mTm^−1^ gradients and a 32‐channel head coil (Nova Medical) in accordance with local ethics procedures. The limiting factors for protocol design were the system SAR limit and eddy current artifacts,^19^ of which the latter were the dominant factor (see discussion). A 3D acquistion with 210 × 210 × 180 mm field‐of‐view and isotropic 1.5 mm voxel size (matrix size 140 × 140 × 120) was used. All phase increments for each ellipse were acquired sequentially before moving to the next ellipse, and 2 seconds of dummy TRs were added at the start of each volume acquisition to establish a steady state. The readout bandwidth was set to 25 kHz and the manufacturer's option to de‐rate the maximum gradient slew rate was enabled, resulting in *TR* = 7 ms for the 256 μs pulse and *TR* = 7.768 ms for the 1024 μs pulse. Partial k‐space acquisition with *NEX* = 0.75 (elliptical k‐space coverage) and parallel imaging (ASSET = 3) was used for a total acquisition time of 17 minutes. Other parameters were the same as for the simulations above. A $B_{1}^{+}$ map was acquired using the manufacturer's Bloch‐Siegert sequence to correct for RF inhomogeneity.^20^, ^21^ All images were motion corrected before further processing.^22^

## 4 RESULTS

### 4.1 Simulations

**Figure 1:**
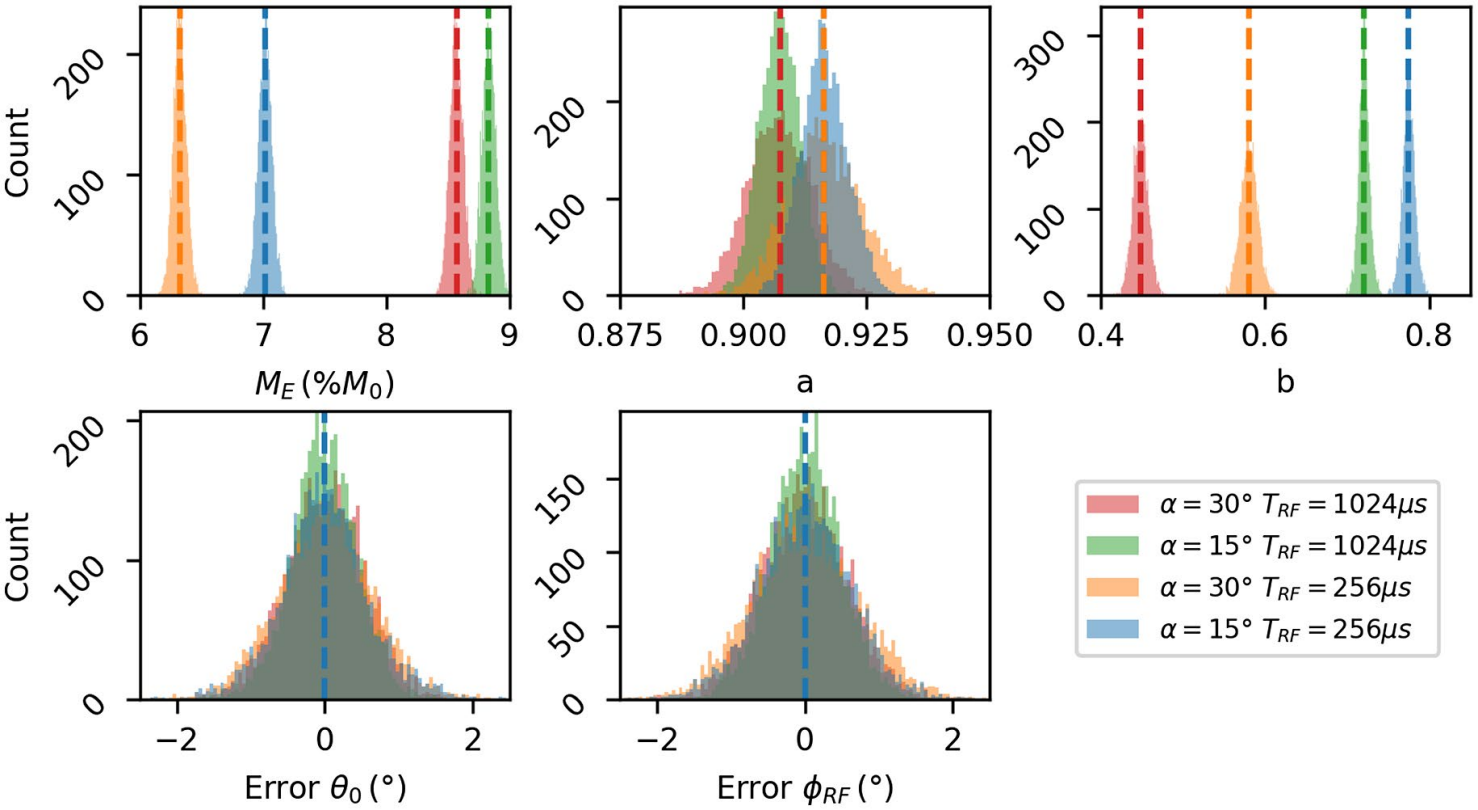
Results of fitting the ellipse parameters *M*_*E*_, *a*, *b* to the Monte Carlo simulations. The parameters for each individual ellipse are shown in the colors given in the legend, and true values are shown with dotted lines

Figure 1 shows the results of the Monte Carlo simulations for the ellipse parameters. Four histograms are present in each subfigure, one for each of the simulated ellipses, and the true values are shown by dotted lines. Parameters *M*_*E*_, *a*, and *b* all have very low bias of less than 1% and coefficient of variation (CoV) of less than 2% for all the ellipses. Note that as the parameter *a* only depends on *T*_2_ and *TR*, there are only two true values and the histograms for 256 μs and 1024 μs pulse‐lengths overlap.

Because the true values of *θ*_0_ and *ϕ*_*RF*_ were chosen randomly (to assess the robustness of the method to off‐resonance and $B_{1}^{+}$ effects), the true value modulus 2*π* was subtracted from the fitted value before plotting, and hence the histograms are centered on zero. Again, the fit is excellent with negligible bias and standard deviations of less than 2%. In the case of *ϕ*_*RF*_, a small number (<1%) of degenerate solutions were found at the +*π*/−*π* boundary. As *ϕ*_*RF*_ is not a parameter of interest for this study, these fitting failures were considered inconsequential, and hence the range of the histogram is restricted to −2^∘^ to 2^∘^.

**Figure 2:**
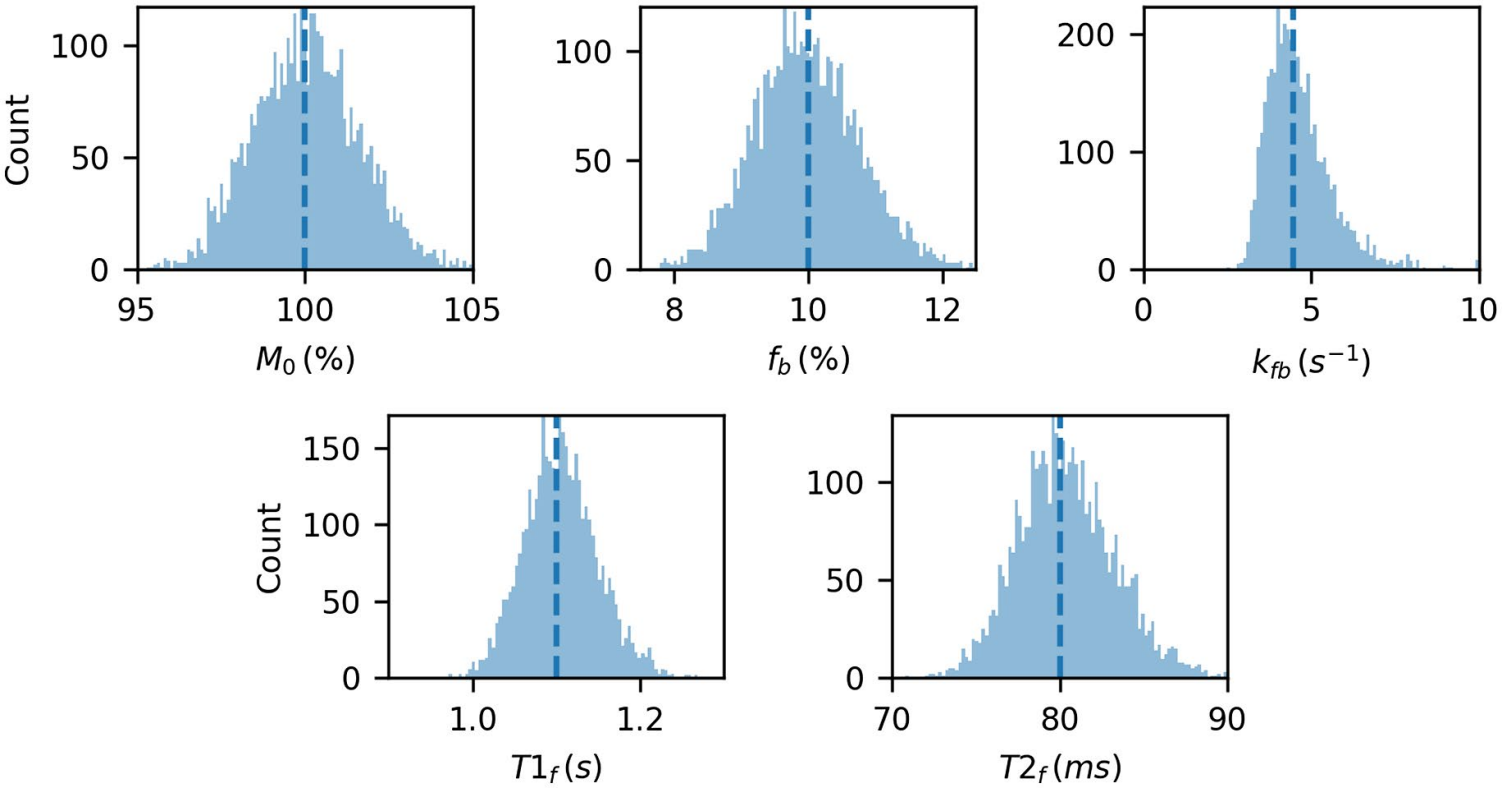
Results of fitting the qMT parameters to the ellipse parameters. *M*_0*f*_, *F*, *T*_1*f*_,*andT*_2*f*_ show the expected normal distribution, but *k*_*bf*_ shows a skewed distribution with a heavy tail toward high values, indicating that this parameter is difficult to fit correctly. True values are indicated with dotted lines

Figure 2 shows the results of fitting the MT parameters to the Monte Carlo simulations. The fits for *M*_0*f*_, *f*_*b*_, *T*_1*f*_ and *T*_2*f*_ showed minimal bias (<1%) and reasonable CoV (<4% except for *f*_*b*_ which was 8%). *k*_*bf*_ shows a skewed distribution, weighted toward higher values, and hence has a CoV of 22% and a bias of 4%.

### 4.2 Experiment

**Figure 3:**
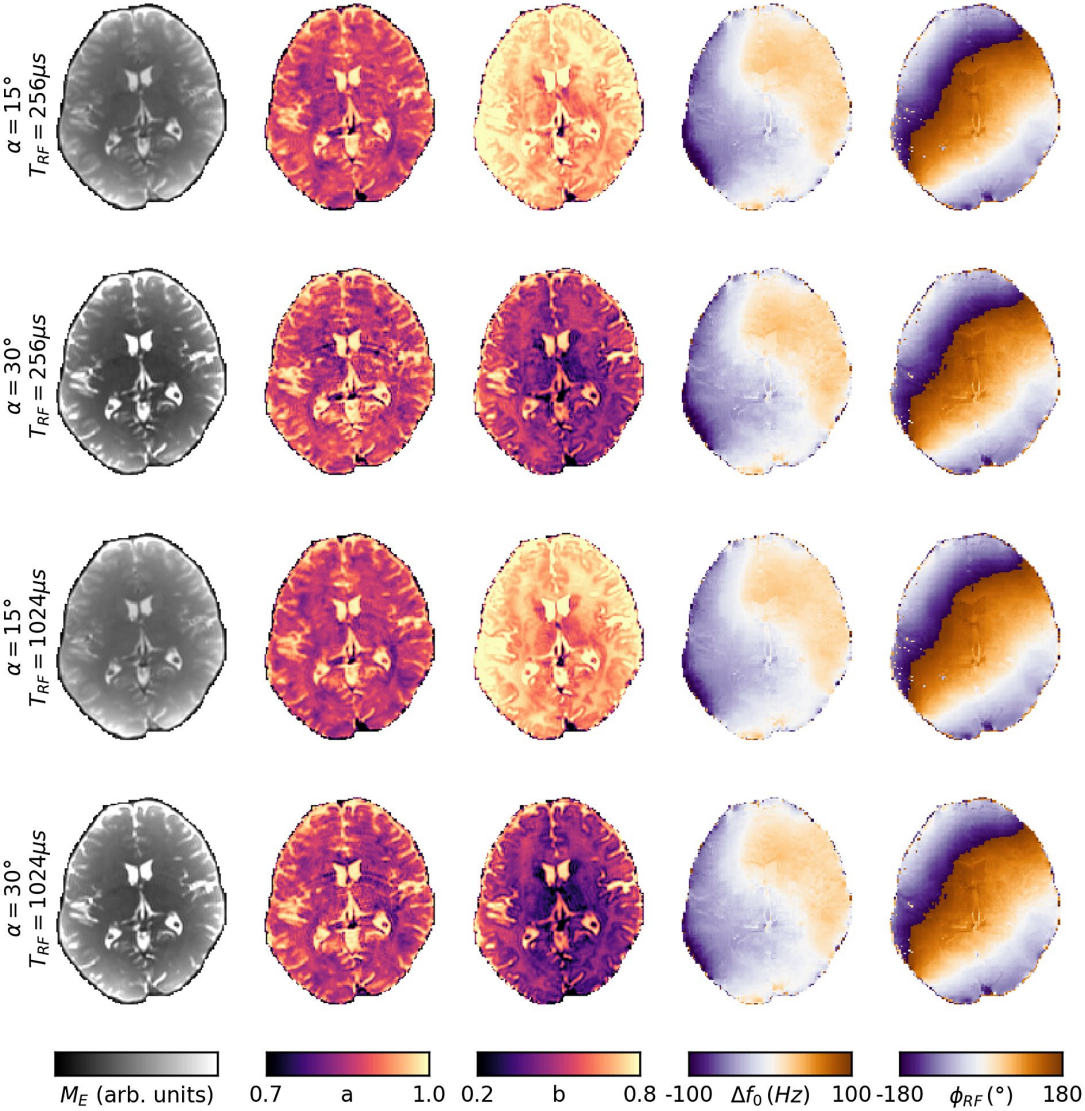
Maps of the ellipse parameters for each MT weighting. The ellipse parameters *M*_*E*_ and *b* change across weightings, while *a* does not. Δ*f*_0_ and *ϕ*_*RF*_ are not of direct interest to this study but are shown for completeness

Figure 3 shows the fitted ellipse parameters in the volunteer for the different MT weightings. The parameters *M*_*E*_ and *b* both show a reduction under increased MT weighting, while there is only a negligible change in *a* due to the different *TR* of the two settings. The off‐resonance frequency (calculated from *θ*_0_) and *B*_1_+ phase are also the same across the scans. While these parameters are not of direct interest to this study, the lack of change indicates excellent scanner stability.

**Figure 4:**
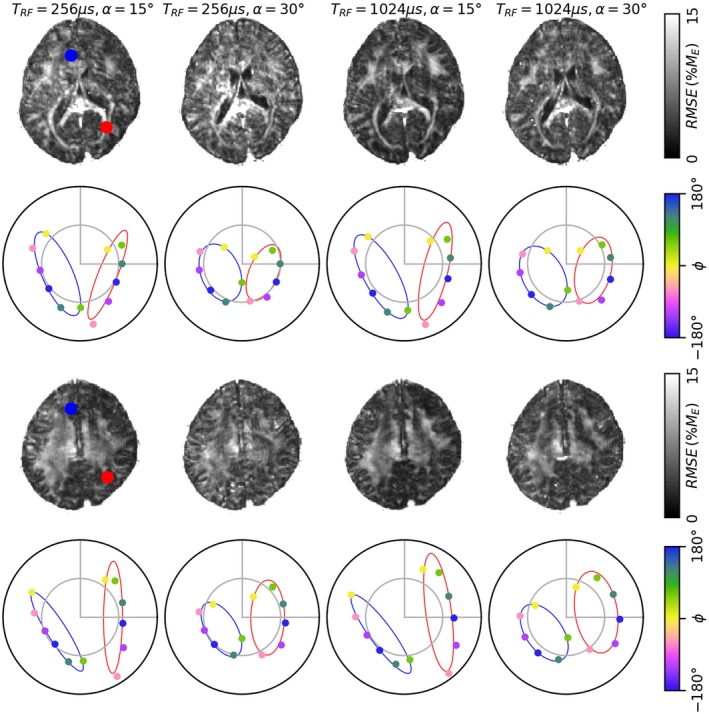
The residuals from fitting each of the four ellipses in two slices (first and third row). In each slice, a low‐ (blue) and high‐ (red) residual voxel are marked and the corresponding fitted ellipses shown (second and fourth rows). The large dots are acquired data (coloured by phase increment) and the line is the fitted ellipse. In low residual voxels, all acquired points on the ellipse, while in high‐residual voxels the *ϕ*_*j*_ = 60^∘^ (light green), does not lie on the ellipse

We observed clear structure in ellipse root‐mean‐square error (RMSE), as shown in Figure 4 (first and third row). This clearly resembles white matter tracts. We plotted the ellipse in selected high‐ and low‐residual voxels (deliberately chosen to lie in opposing halves of the complex plane for clarity) to further illustrate the behavior (second and fourth row).

**Figure 5:**
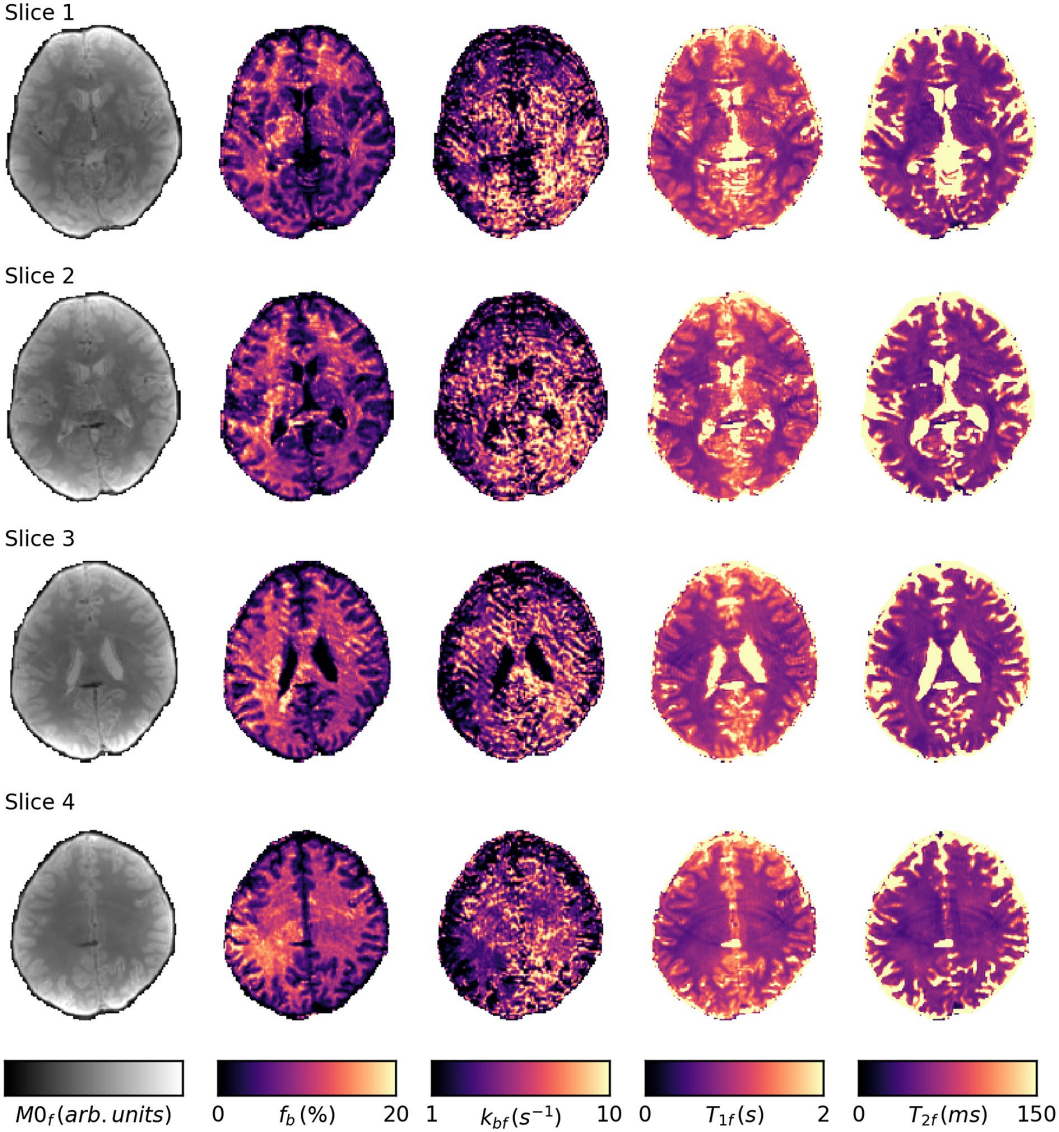
Maps of the fitted quantitative MT parameters through four slices of the brain. The exchange parameter *k*_*bf*_ shows a large number of fitting failures where the value has reach the upper fitting bound; however, this does not appear to impact the fitting of the other parameters

Finally, Figure 5 shows maps of the MT parameters. The maps of *M*_0*f*_, *f*_*b*_, *T*_1*f*_ and *T*_2*f*_ show good fitting quality. In contrast, the map of *k*_*bf*_ displays a large number of fitting failures (bright voxels). A large bilateral ROI was drawn in WM and gave the following average values: *f*_*b*_ = 11.3 ± 2.7%, *T*_1*f*_ = 794 ± 126 ms, *T*_2*f*_ = 54 ± 19 ms, and finally *k*_*bf*_ = 5.0 ± 2.4 s^−1^.

## 5 DISCUSSION

The results above demonstrate that it is possible to extract *T*_1_, *T*_2_, proton density, bound‐pool fraction, and exchange rate information from SSFP data using an elliptical signal model, with no additional measurement of the apparent *T*_1_. A *B*_1_^+^ map is still required to calculate the local flip angle. Several confounds and difficulties present themselves with the current approach—in particular, the frequency distribution of white matter protons, the lengthy acquisition time, lack of precision in quantifying the exchange rate, and an inability to probe the bound‐pool lineshape.

The most significant issue with the current work is the increased residual for fitting the ellipses in white matter, which indicates that the above model does not fully explain the behavior of the SSFP signal in such regions. White matter is known to exhibit multiple water components with different *T*_2_, which are generally attributed to free water and water trapped in myelin bilayers.^18^, ^23^ Indeed, Shcherbakova et al discussed how a two component system could be modeled with the ellipse formalism, but importantly only showed simulation results and did not discuss exchange effects between the pools.^8^ They suggested that within typical ranges of TR and flip angle the expected impact of the frequency distribution on PLANET is minimal. Similarly, our observed deviations from the ellipse appear small. Although the RMSE can approach 15% of *M*_*E*_ in WM, the majority of points exhibited an excellent fit and only a single outlier phase increment was observed in most voxels. The exact phase increment varied with off‐resonance frequency across the brain.

Previously, Miller et al discussed at length the asymmetries of the SSFP signal profile in white matter, but did not use the ellipse formalism.^9^, ^10^ We argue that the residual maps shown in Figure 3 are similar to Figure 3 of,^10^ and that if translated to the complex plane the asymmetry profiles would resemble the ellipses of Figure 4. Hence we conclude that the increased residuals displayed for fitting the ellipse in white matter are consistent with previous literature and can be attributed to the frequency distribution of protons in the myelin water pool. Quantifying the impact of the frequency distribution on the subsequent MT quantification would require modeling both MT and the frequency distribution, which is beyond the scope of this work.

The acquisition time and exchange‐rate issues are linked. There are two reasons for the relatively lengthy acquisition time described here, relative to the modest voxel size: the number of acquired images and the long *TR*. Our protocol required 24 separate SSFP images to be acquired with different flip angles, pulse lengths and phase increments, in order to use the two‐stage fitting process where we first fit the ellipse parameters and then the MT parameters. However, there are only seven parameters in the final set (*M*_0_, *f*_*b*_, *k*_*bf*_, *T*_1*f*_, *T*_2*f*_, off‐resonance, and *B*_1_^+^ phase), and so the current protocol is highly redundant. In principle, fewer SSFP images would be required if the MT‐ellipse model were fitted directly to the data, which could yield a significant speed‐up. This was beyond the scope of this paper as we wished to illustrate the ellipse behavior in WM, which would be hidden by such a model. An additional benefit to a shorter protocol would be increased robustness against subject motion and frequency drift effects.^24^

We found that a relatively long *TR* was required to avoid eddy current artifacts that affected the different phase increment images in unpredictable ways if the full gradient performance of our system was exploited. These artifacts are not usually present in traditional SSFP images acquired with a 180^∘^ phase increment and standard Cartesian k‐space ordering because any eddy currents cancel out in successive *TR*s.^19^ However, in this work, images are deliberately acquired with phase increments that do not lead to such cancellation (see Supporting Information Figure S1). As noted in the methods section, for the purposes of this paper, we de‐rated the gradient slew rate to remove the artifacts, which almost doubled the *TR*.

Shortening the *TR* could also improve the fitting of *k*_*bf*_, which has been shown to contain clinically useful information.^25^ We found that the cost function, described by Equation 11, narrows in the exchange‐rate direction when *TR* is shortened, implying that *k*_*bf*_ would then be easier to fit (see Supporting Information Figure S2). Due to our long *TR*, we did not consider the effects of finite RF pulse duration on our *T*_2*f*_ estimates, but this may become a significant effect with shorter *TR*.^26^

In this paper, a constant value of *T*_2*b*_ across the brain was assumed.^5^, ^13^ However, data from classic gradient‐echo qMT experiments indicate the apparent *T*_2*b*_ varies across brain when using a Super‐Lorentzian lineshape.^27^ However, due to the use of on‐resonance RF pulses in this method, the tails of the lineshape are not queried at all and hence it is not possible to fit for *T*_2*b*_. It may be possible to query the bound‐pool lineshape by acquiring additional images with a multi‐band excitation pulse,^28^ but this was beyond the scope of this article.

## 6 CONCLUSION

We have demonstrated that MT parameters can be extracted from the SSFP signal ellipse. This method does not require an additional *T*_1_ map as this information comes directly from the model. The method is robust against SSFP banding artifacts because off‐resonance effects are directly incorporated into the model. The acquired data showed deviations from theory in white matter which can be attributed to frequency distribution effects.

## Supporting information


**FIGURE S1** Example raw images acquired at the minimum possible *TR* for our system. In addition to the SSFP bands (which are not artifacts in the context of this work), there are an additional zipper‐type eddy current artifacts present when *ϕ* ≠ 180^∘^ which are marked by arrows. These artifacts change position depending on the phase increment value, and only disappeared when *TR* was increased significantly
**FIGURE S2** Contour plots of the MT fitting cost function in the *f*_*b*_, *k*_*bf*_ plane. The red dot marks the ground truth value. Reducing *TR* steepens the contours in the *k*_*bf*_ direction, which would make fitting the true value easier, but does not significantly change the shape of the contours in the *f*_*b*_ direction
