# Supplementary material for: Magnetization transfer and frequency distribution effects in the SSFP ellipse
Source: Magn Reson Med. 2019 Dec 24;84(2):857–65. doi: 10.1002/mrm.28149 (PMC7216875; doi:10.1002/mrm.28149)
Supplement: Supplementary file 1 — FIGURE S1 Example raw images acquired at the minimum possible TR for our system. In addition to the SSFP bands (which are not artifacts in the context of this work), there are an additional zipper‐type eddy current artifacts present when ϕ ≠ 180∘ which are marked by arrows. These artifacts change position depending on the phase increment value, and only disappeared when TR was increased significantly FIGURE S2 Contour plots of the MT fitting cost function in the f b, k bf plane. The red dot marks the ground truth value. Reducing TR steepens the contours in the k bf direction, which would make fitting the true value easier, but does not significantly change the shape of the contours in the f b direction [file MRM-84-857-s001.pdf]

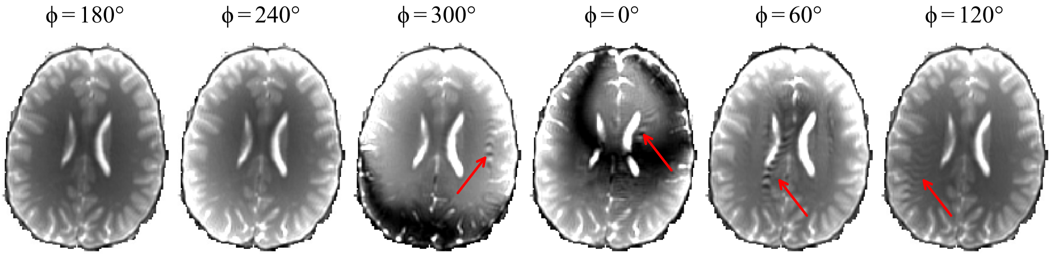

FIGURE S1 Example raw images acquired at the minimum possible *TR* for our system. In addition to the SSFP bands (which are not artefacts in the context of this work), there are an additional zipper-type eddy current artefacts present when $\phi \neq 180°$ which are marked by arrows. These artefacts change position depending on the phase increment value, and only disappeared when *TR* was increased significantly.

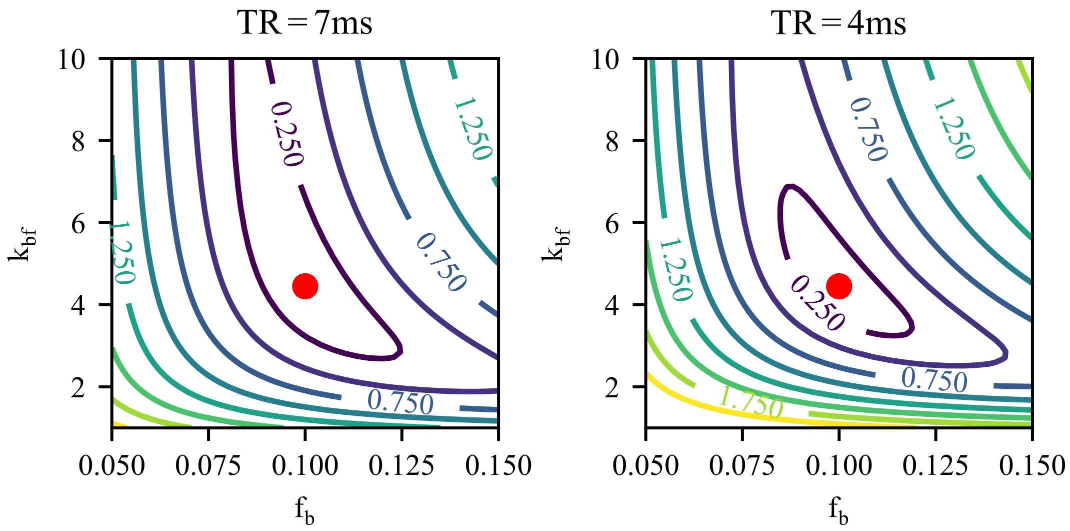

FIGURE S2 Contour plots of the MT fitting cost function in the f_b, k_{bf} plane. The red dot marks the ground truth value. Reducing *TR* steepens the contours in the k_{bf} direction, which would make fitting the true value easier, but does not significantly change the shape of the contours in the f_b direction.
